# Sulfur dioxide exposure reduces the quantity of CD19^+^ cells and causes nasal epithelial injury in rats

**DOI:** 10.1186/s12995-018-0205-x

**Published:** 2018-07-27

**Authors:** Ruonan Chai, Hua Xie, Junli Zhang, Zhuang Ma

**Affiliations:** Department of Respiratory Medicine, General Hospital of Shenyang Military Command, No. 83 Wenhua Road, Shenhe District, Shenyang, 110016 China

**Keywords:** Reactive airway, Dysfunction syndrome, Asthma, SO_2_, CD19

## Abstract

**Background:**

Reactive airway dysfunction syndrome (RADS), also called irritant-induced asthma, is a type of occupational asthma that can occur within a very short period of latency. The study sought to investigate the influence of sulfur dioxide (SO_2_) exposure on CD19+ cells and nasal epithelial injury.

**Methods:**

We investigated the effects of SO_2_ on CD19 expression and morphological changes of nasal epithelia in rats. In the study, 20 rats were randomly divided into the SO_2_ exposure group that were exposed to 600 ppm SO_2_, 2 h/day for consecutive 7 days, and the control group that were exposed to filtered air).

**Results:**

Inhalation of high concentration of SO_2_significantly reduced CD19 expression at both the mRNA transcript and protein levels, and reduced the percentages of CD19^+^ cells and CD19^+^/CD23^+^ cells in the nasal septum. However, inhalation of high concentration of SO_2_ did not affect immunoglobulin (Ig) G, IgA and IgE levels in the serum and nasal septum. More importantly, SO_2_ exposure also caused mild structural changes of the nasal septum.

**Conclusion:**

Our results reveal that inhalation of a high concentration of SO_2_ reduces CD19 expression and causes structural change of the nasal septum in rats.

## Highlights

- SO_2_ inhalation reduced the CD19 expression in nasal septum
- SO_2_ exposure decreased the percentage of CD19+ and CD19+/CD23+ cells
- SO2 inhalation does not affect the IgG, IgA and IgE levels

## Background

Reactive airway dysfunction syndrome (RADS), also called irritant-induced asthma, is a type of occupational asthma that can occur within a very short period of latency [1]. With intensive studies on the pathogenesis of asthma, a deep insight into RADS has been gained [2]. RADS is characterized clinically by asthma-like symptoms including cough, wheezing, chest tightness, and breathlessness. The symptoms of RADS usually occur within 24 h after exposure to high amounts of harmful gases [3]. RADS shares no features of immunology and allergy, which is distinct from classic asthma [4]. However, clinical manifestations of both RADS and asthma are very similar and both share common characteristics, especially airway hyperresponsiveness [5]. Therefore, RADS is thought as a type of occupational asthma, or an adult-onset asthma [6]. The exact cause of RADS is not yet known, but the syndrome is considered to be uncommon and recognized in less than one-fifth of workers with “occupational asthma” [7].

Sulfur dioxide (SO_2_) emissions are mainly produced from industrial processes, including metal extraction from ores, power plants and refineries. Owing to its rotten-egg odour, sulfur dioxide can be noted at levels between 0.3 and 5 ppm (ppm). Exposure to levels as low as 0.1 to 0.5 ppm can cause bronchoconstriction in humans with asthma. Especially, the influence of exposure to high concentrations of SO_2_ for a short time has been documented [8–10]. SO_2_ accounts for the major proportion of smoke of gunpowder, and its concentration is very high, which might cause respiratory dysfunction syndrome. In addition, SO_2_ tank leaks or smoke from mine blast can also expose humans to high concentrations of SO_2_ for a short time.

B-lymphocyte antigen CD19, also known as CD19, is found on the surface of B-cells, a type of white blood cell. CD19 plays a physiological function in establishing intrinsic B cell signaling thresholds through modulating both B cell receptor (BCR)-dependent and independent signaling [11]. CD19 plays an important role in immunoglobulin-induced activation of B cells [12]. CD19 deficiency causes hyporesponsiveness to transmembrane signals, and weak T cell-dependent humoral responses [13]. CD23, also known as Fc epsilon RII, or FcεRII, is important in regulating IgE levels. CD32 is a surface receptor protein, which has a low-affinity for IgG antibodies and down-regulates antibody production in the presence of IgG. Immunoglobulins, including IgG, IgA and IgE, are large, Y-shaped proteins produced by plasma cells to neutralize pathogens such as pathogenic bacteria and viruses. However, the effects of SO_2_ on CD19 and immunoglobulin expression have not been reported. In this study, we exposed rats to a high concentration of SO_2_, and determined changes in the percentage of CD19^+^ cells.

## Methods

### Animals and reagents

Twenty male Sprague Dawley rats (weighing 180–200 g each) were purchased from Liaoning Changsheng Biotechnology (Shenyang, China). SO_2_ gas (purity: 99.9%) was purchased from Beijing Ya-nan Gas Scientific and Technology (Beijing, China). Anti-CD19-FITC monoclonal antibody and goat anti-rat IgG, IgA and IgE antibodies were purchased from Abcam Corp. Ltd. (Abcam, UK). Anti-CD32 antibody and goat ABC staining system were purchased from Santa Cruz Biotechnology (Santa Cruz, CA, USA). Rat IgG, IgA and IgE ELISA kit was purchased from eBioscience Corp. (USA). TRIzol reagent was purchased from Takara Corp. (USA). Experiment protocols strictly complied with the Laboratory Animal Administration Rules.

### Exposure chamber

SO_2_ exposure was conducted as previously described with slight modification [14, 15]. The device consists of SO_2_ source, air pump, intake port, SO_2_ chamber, SO_2_ detector and several connective tubes/valves. SO_2_ was diluted with fresh air in the intake port to yield desired SO_2_ concentrations. SO_2_ was delivered to animals via a tube positioned at the upper level of the chamber and distributed homogeneously via a fan in each chamber. The concentration of SO_2_ was determined in a real-time manner by SO_2_ sensor (JSA5-SO_2_ sensor, Shenzhen Ji-shun-an Technology Co., Ltd., China). The concentration of SO_2_ in the chamber was selected by adjusting the valve between the intake port and SO_2_ chamber according to the quantitative value of the SO_2_ sensor.

### Exposure protocol

Twenty rats were randomly divided into the SO_2_ exposure group and the control group (10 in each group). The rats in the SO_2_ exposure group were placed into the exposure chamber (600 ppm SO_2_, 2 h/day) for consecutive 7 days, while rats in the control group were exposed to filtered air in another identical chamber for the same duration. All rats were allowed free access to food and water. After 7-day exposure, the tail vein blood from each rat was taken. The blood was kept at room temperature for 2 h, and clarified by centrifugation to obtain serum. Then, the rats were sacrificed with quick decapitation. The nasal septum was removed and washed with phosphate buffered saline (PBS).

### Real time quantitative reverse Transcrption PCR (qRT-PCR)

The nasal septum was collected and total RNA was prepared with Trizol reagent according to the manufacturer’s instructions. Real time qRT-PCR was performed using a sequence detection system (ABI PRISM 7000; Applied Biosystems, Life Technologies,Grand Island, NY, USA), with a commercially available kit (SYBR PrimeScriptRT-PCR Kit, TaKaRa Biotechnology). The reactions were performed at least three times independently. The cycle threshold analysis of all samples was set automatically by the ABI PRISM 7000 software. The mRNA expression of the indicated gene was normalized against β-actin. The annealing temperature for β-actin and CD19 was 60 °C.

The sequences of the primers (Sangon, Shanghai, China) used in the study were as follows:

- *β-actin* (211 bp) sense: 5’-CCTCTATGCCAACACAGTGC-3′, and antisense: 5’-GTACTCCTGCTTGCTGATCC-3′, and *CD19* (273 bp) sense: 5’-ATGTGGGTTTGGGGGTCTC-3′, and antisense: 5’-AGGGTCGGTCATTCGCTTC-3′.

### Western blotting assays

Western blot analysis was performed of cellular lysates of the nasal septum as described previously [16]. Briefly, proteins were resolved by sodium dodecyl sulfate–polyacrylamidegel electrophoresis (SDS-PAGE, 10% separating, 5% stacking) and transferred to PVDF membranes (Millipore, USA). The membranes were blocked by 5% defat milk in PBS containing 0.1% Tween 20. Thereafter, the membranes were incubated with monoclonal antibody specific for rat CD19 (dilution 1:5000) at 4 °C overnight. Anti-mouse secondary antibody (dilution 1:5000) was added to membranes and incubated at 37 °C for 45 min. Protein signal was amplified and visualized via chemiluminescence using the ECL detection system and Hyperfilm ECL autoradiography film (Amersham Pharmacia Biotech, Inc.). Protein expression was normalized against β-actin. Images were quantified using the Labworks v3.0.2 image scanning and analysis software (Gel-Pro-Analyzer).

### Elisa

The levels of IgG, IgA and IgE in the plasma and nasal septum were measured using commercially available ELISA kit according to the manufacturer’s instructions and calculated by generating a standard curve using standard proteins and analyzed using Curve Expert 1.3 Software.

### Flow cytometry

The nasal septum was minced and digested with 0.04% collagenase IV in DMEM at 37 ^o^Cfor 1 h. Digested cells were washed with PBS to remove collagenase. The tissues were homogenized and centrifuged. Cells were pooled and suspended in PBS with 1% BSA at a density of 1 × 10^7^ cells/mL. Anti-CD16 and anti-CD32 antibodies were added at a concentration of 1 μg/10^6^ cells to block Fc receptors by incubation on ice for 10 min. Cells were washed with PBS and stained with 1 μg anti- CD19-FITC and anti-CD23-PE antibodies at 4^o^C for 30 min in the dark. The cells were washed again and analyzed by flow cytometry.

### Histological analysis

The nasal septum was fixed in 4% formaldehyde. The fixed tissue samples were dehydrated in graded ethanol, embedded in paraffin. Each paraffin block was sectioned into 5-μm-thick slices, which were then dewaxed in xylene, rehydrated in gradient alcohols and rinsed with distilled water. Each section placed on glass slide was stained with hematoxylin and eosin (H&E) and double-blindly evaluated under light microscope by an experienced histologist.

### Statistical analysis

All values were expressed as mean ± standard deviation (SD). Data analysis was performed using Student’s *t* test. *P* < 0.05 was considered statistically significant.

## Results

### SO_2_ inhalation reduces CD19 expression in the nasal septum

**Fig. 1:**
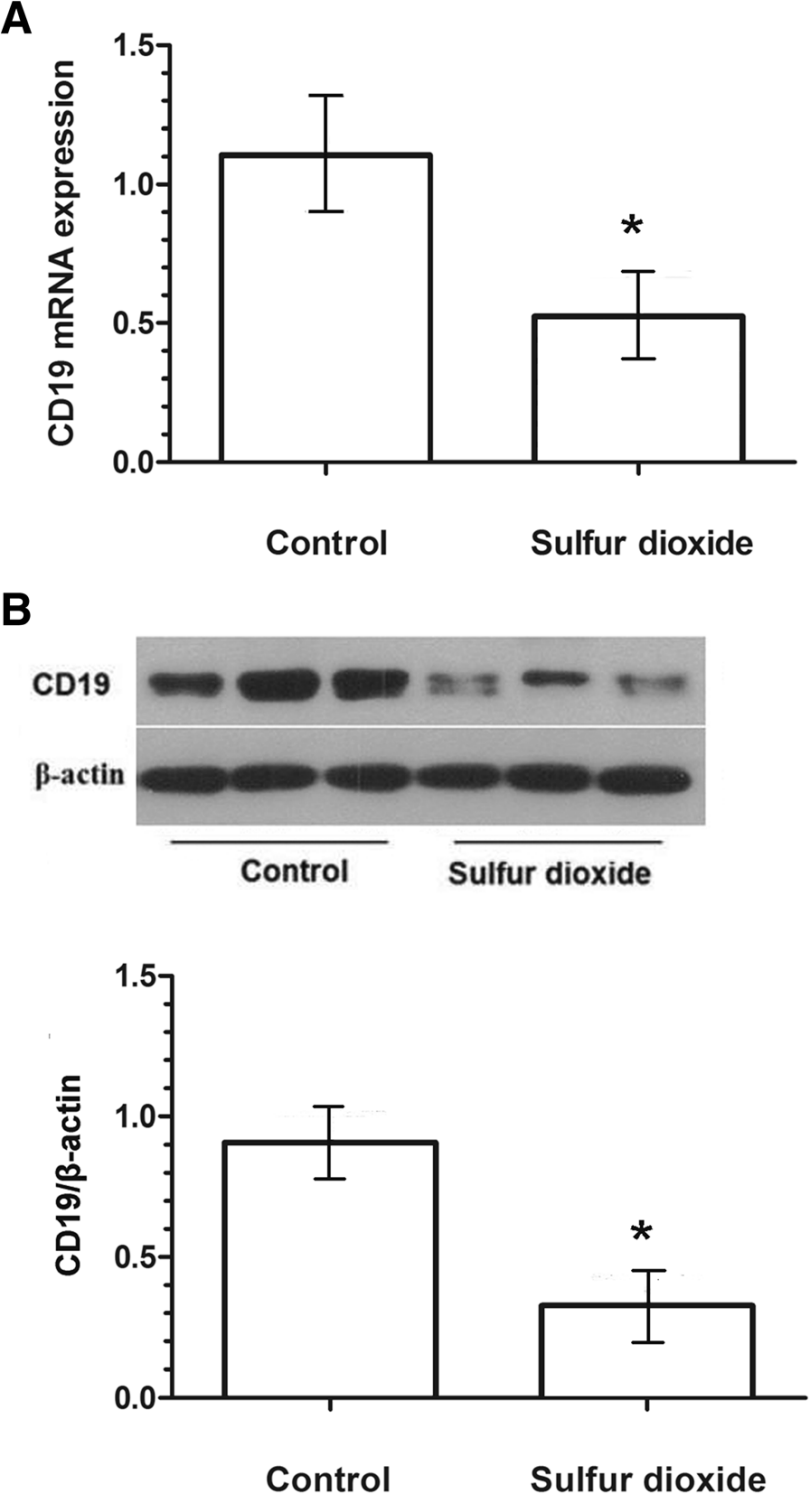
SO_2_ inhalation reduces the expression of CD19 expression in the nasal septum. **a**: mRNA expression; **b**: protein expression. *P* < 0.05 compared with controls

The rats were healthy before the experiment. When exposed to high concentrations of SO_2_, rats became inactive and curled together. No obvious symptoms and weight loss were observed after the exposure. The expressions of both CD19 mRNA transcripts and protein were significantly decreased in SO_2_ exposed rats when compared with the control group (all *P* < 0.05) (Fig. 1a and b).

### SO_2_ exposure significantly decreases the percentage of CD19^+^ and CD19^+^/CD23^+^ cells in the nasal septum

**Fig. 2:**
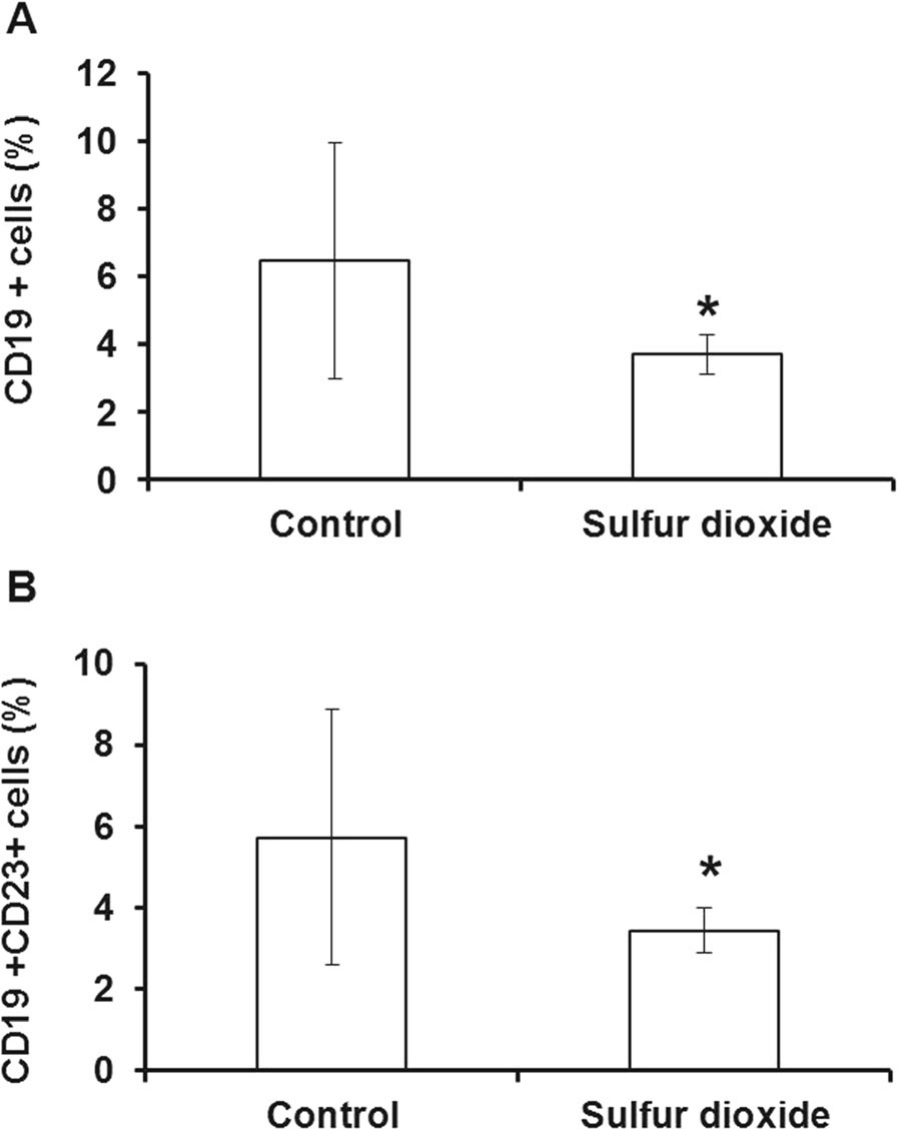
SO_2_ inhalation reduces the percentage of CD19^+^ (**a**) and CD19^+^/CD23^+^ cells (**b**) in the nasal septum. P < 0.05 compared with controls

Flow cytometry was applied to determine the percentage of CD19^+^ and CD19^+^ CD23^+^ cells in the nasal septum. The results showed that the proportion of both CD19^+^ cells (6.49 ± 3.48% vs. 3.71 ± 0.57% *P* < 0.05), and CD19^+^/CD23^+^ cells (5.74 ± 3.14% vs. 3.45 ± 0.54%, *P* < 0.05) were significantly decreased after SO_2_ exposure compared with the control group (Fig. 2a and b).

### SO2 inhalation does not affect IgG, IgA and IgE levels in the plasma and nasal septum

**Fig. 3:**
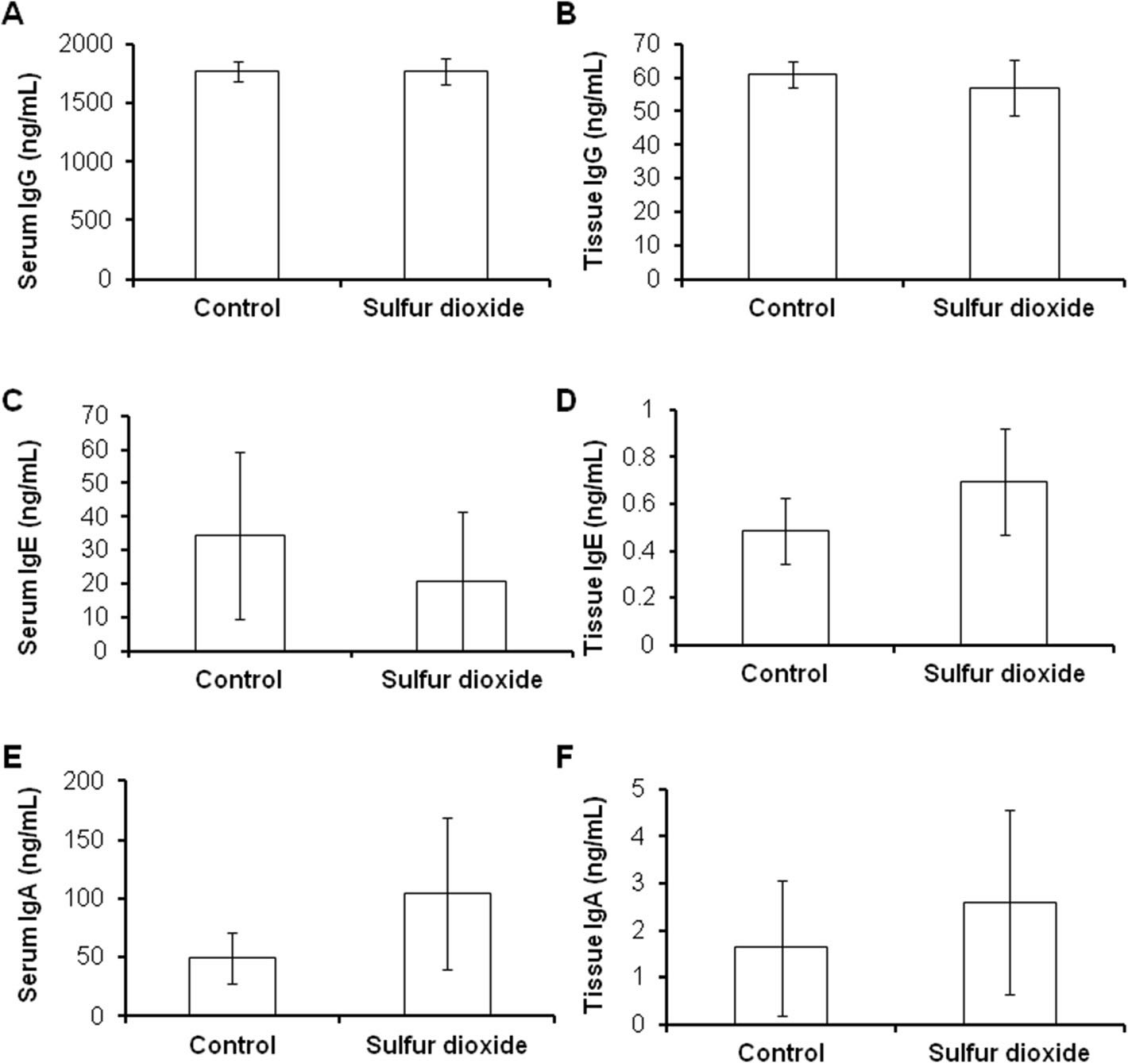
The representative figure showed that SO_2_ inhalation did not affect IgG levels in the serum and nasal septum. **a**: IgG level in the serum; **b**: IgG level in the nasal septum; **c**: IgE level in the serum; **d**: IgE level in the nasal septum; **e**: IgA level in the serum; **f**: IgA level in the nasal septum

Since B lymphocyte plays an important role in activation of antibody production, we measured IgG levels in the plasma and nasal septum after SO_2_ exposure by ELISA to determine whether antibody production was influenced by the down-regulation of CD19 upon SO_2_ exposure. However, no differences were observed in IgG, IgA and IgE levels in both the plasma and nasal septum between the two groups (Fig. 3).

### Histopathological changes after SO_2_ exposure

**Fig. 4:**
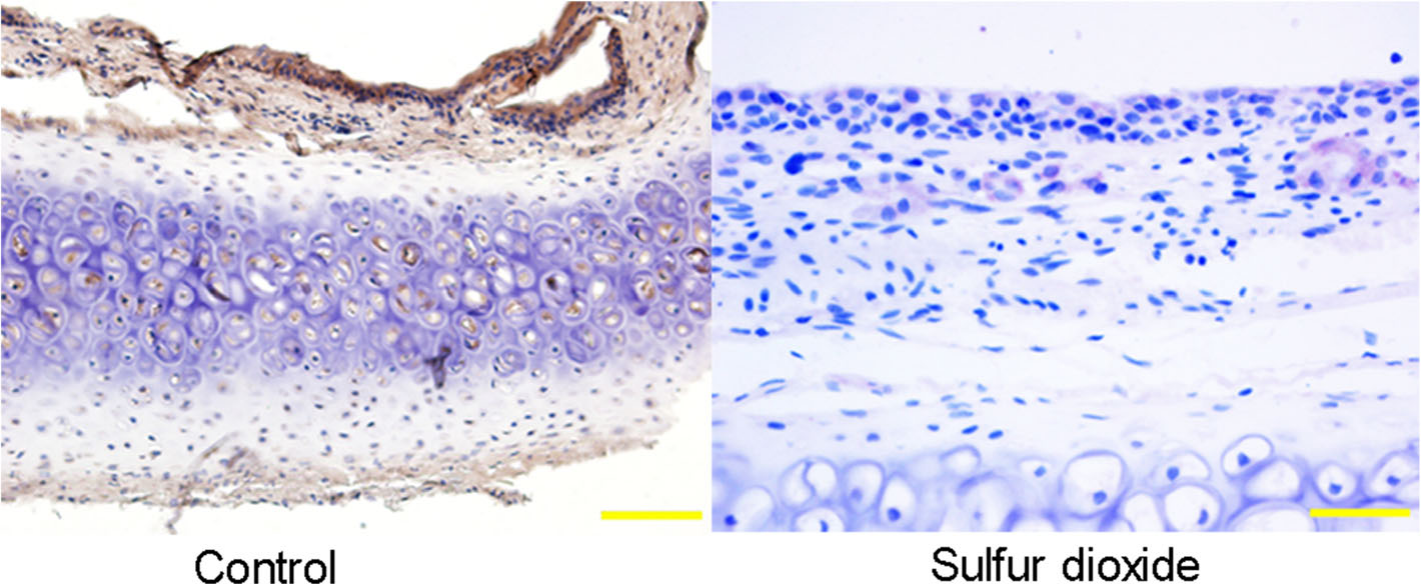
The structure of the nasal septum of SO_2_ exposed rats looked blurred, indicating slight structural changes

Histopathological studies showed that the structure of nasal septum was damaged and the boundary for different layers was blurry in exposed rats, while the structure of layers in the control group was normal. By contrast, no apparent alveolar hemorrhage and lymphocytes infiltration were observed in both two groups (Fig. 4).

## Discussion

In this study, we showed that SO_2_ exposure not only reduced CD19 expression, but also caused nasal epithelial injury. Considering that SO_2_ is one of the major components of RADS-related air pollutants, this study might provide important implications for the pathogenesis of RADS.

Irritative chemical gases may firstly cause injury of airway epithelial cells, followed by inflammation, edema and blockage of bronchioles, which are similar to chemical trachitis and bronchitis [17]. RADS has a longer course compared with chemical trachitis and bronchitis. In most cases, lung function in RADS can recover 3–6 months after evacuation of irritating environment. RADS likely progresses into chronic disease, if symptoms persist for more than 6 months [18]. The membrane potential of the cells is altered in high cation condition, whereas changes of membrane potential can elicit the increase of oxygen radicals in cell respiratory burst, which is involved in the inflammatory process [19, 20].

SO_2_ is a major composition in air pollutants and is characteristically pungent and suffocating. After exposure, the physiological function of the respiratory system is impaired, accompanied by weakened elasticity of pulmonary alveoli and decreased lung function, which resulted in trachitis, bronchial asthma and emphysema, etc. [21, 22]. In 2012, we obtained a fund to investigate the effect of smoke on the human respiratory system in the battlefield environment. After the analysis of the composition of the smoke, we found that SO_2_ was the major component of gunpowder smoke, and the concentration was very high. Therefore, we designed this experiment to simulate the respiratory dysfunction syndrome caused by smoke inhalation by soldiers or civilians in a war environment and investigate its pathogenesis. In addition, in a peaceful environment, SO_2_ tank leaks or smoke from mine blast can also expose humans to high concentrations of SO_2_ for a short time. Therefore, the investigation of exposure to high concentration of SO_2_ is of practical significance, although 600 ppm is a lethal concentration for humans. CD19 is an important marker of B lymphocytes and plays a key role in B cell signal transduction, activation, differentiation and production of antibody [23]. In this study, the effects of SO_2_ exposure on CD19 expression and the percentage of CD19^+^ cells were investigated after inhalation of high concentration of SO_2_. This study revealed an important mechanism underlying SO_2_ exposure-induced RADS.

Our results showed that multiple inhalation exposures to high concentration of SO_2_ could result in nasal septum injury, but not death in rats. However, potential influence of high concentration of SO_2_ on the lungs and bronchoalveolar lavage should be investigated in future study. Additionally, the injury in our study is different from that of bronchial asthma [24]. In classic humoral immunity, bronchial asthma is manifested by increase of immunoglobulin (for example IgG) and CD19^+^ cell counts. Whereas the percentage of CD19^+^ cells and CD19^+^/CD23^+^ cells in nasal mucosa tissues demonstrate a significant decrease compared with the control in our model. Real time qRT-PCR and Western blot analysis further demonstrated a decreased expression of CD19 after inhalation of high concentration of SO_2_. Moreover, the levels of IgG, IgA and IgE were not influenced. But histopathological studies showed that the structure of the nasal septum appeared blurred.

In summary, multiple SO_2_ exposures induce structural changes of the nasal septum and down-regulates the expression of CD19 in the rat. Further studies are required to explore the molecular mechanism after SO_2_ exposure and the role of CD19.

## References

[1] Doshi V, Kham N, Kulkarni S, Kapitan K, Henkle J, White P. R-134a (1,1,1,2-Tetrafluoroethane) inhalation induced reactive airways dysfunction syndrome. Am J Therap. 2016;23(3):e969–71. 10.1097/MJT.0000000000000124 25137406

[2] Brooks SM (2016). Then and now reactive airways dysfunction syndrome. J Occup Environ Med.

[3] Shakeri MS, Dick FD, Ayres JG (2008). Which agents cause reactive airways dysfunction syndrome (RADS)? A systematic review. Occup Med.

[4] Murphy DM, O'Byrne PM (2010). Recent advances in the pathophysiology of asthma. Chest.

[5] Brooks SM, Bernstein IL (2011). Irritant-induced airway disorders. Immunol Allergy Clin.

[6] Muñoz X, Cruz MJ, Bustamante V, Lopez-Campos JL, Barreiro E (2014). Work-related asthma: diagnosis and prognosis of immunological occupational asthma and work-exacerbated asthma. J Invest Allerg Clin.

[7] SM B (2013). Reactive airways dysfunction syndrome and considerations of irritant-induced asthma. J Occup Environ Med.

[8] Guarnieri M, Balmes JR (1996). Outdoor air pollution and asthma. Food Chem Toxicol.

[9] Wang XB, Du JB, Cui H (2014). Sulfur dioxide, a double-faced molecule in mammals. Life Sci.

[10] Charan NB, Myers CG, Lakshminarayan S, Spencer TM (1979). Pulmonary injuries associated with acute sulfur dioxide inhalation. Am Rev Respir Dis.

[11] Bai X, Huang L, Niu L, Zhang Y, Wang J, Sun X (2016). Mst1 positively regulates B-cell receptor signaling via CD19 transcriptional levels. Blood Adv..

[12] Poole JA, Mikuls TR, Duryee MJ, Warren KJ, Wyatt TA, Nelson AJ (2017). A role for B cells in organic dust induced lung inflammation. Respir Res.

[13] Abe M, Wang Z, De CA, Thomson AW (2015). Plasmacytoid dendritic cell precursors induce allogeneic T-cell hyporesponsiveness and prolong heart graft survival. Am J Transpla.

[14] Bai J, Meng Z (2005). Effects of sulfur dioxide on apoptosis-related gene expressions in lungs from rats. Regul Toxicol Pharmacol.

[15] Li R, Meng Z, Xie J (2008). Effects of sulfur dioxide on the expressions of EGF, EGFR, and COX-2 in airway of asthmatic rats. Arch Environ Con Tox.

[16] Qin G, Meng Z, editors. Effect of Sulfur Dioxide Inhalation on CYP2B1/2 and CYP2E1 in Rat Liver and Lung. Inhal Tox. 2008;581–88. 10.1080/08958370600686341 16717030

[17] Cooney DJ, Hickey AJ (2011). Cellular response to the deposition of diesel exhaust particle aerosols onto human lung cells grown at the air-liquid interface by inertial impaction. Tox In Vitro.

[18] Du CL, Wang JD, Chu PC, Guo YL (2010). Acute expanded perlite exposure with persistent reactive airway dysfunction syndrome. Ind Health.

[19] Schreiber R (2005). Ca2+ signaling, intracellular pH and cell volume in cell proliferation. J Membrane Biol.

[20] Xie Y, Zhuang ZX (2001). Chromium(VI)-induced Production of Reactive Oxygen Species, Change of Plasma Membrane Potential and Dissipation of Mitochondria Membrane Potential in Chinese Hamster Lung Cell Cultures. Biomed Environ Sci.

[21] Winkler T, Suki B (2011). Emergent structure-function relations in emphysema and asthma. Crit Rev Biomed Eng.

[22] Kahana LM, Aronovitch M (1968). Pulmonary surface tension after sulfur dioxide exposure. Am Rev Respir Dis.

[23] Brynjolfsson SF, Mohaddes M, Kärrholm J, Wick MJ (2017). Long-lived plasma cells in human bone marrow can be either CD19+ or CD19. Blood Adv.

[24] Ukena D, Fishman L, Niebling WB (2008). Bronchial asthma: diagnosis and long-term treatment in adults. Dtsch Rztebl Int.

